# The Response of Vocal Fold Fibroblasts and Mesenchymal Stromal Cells to Vibration

**DOI:** 10.1371/journal.pone.0030965

**Published:** 2012-02-16

**Authors:** Joel Gaston, Beatriz Quinchia Rios, Rebecca Bartlett, Craig Berchtold, Susan L. Thibeault

**Affiliations:** 1 Department of Biomedical Engineering, University of Wisconsin Madison, Madison, Wisconsin, United States of America; 2 Division of Otolaryngology Head and Neck Surgery, Department of Surgery, University of Wisconsin Madison, Madison, Wisconsin, United States of America; University of Massachusetts Medical, United States of America

## Abstract

Illumination of cellular changes caused by mechanical forces present within the laryngeal microenvironment may well guide strategies for tissue engineering the vocal fold lamina propria. The purpose of this study was to compare the response of human vocal fold fibroblasts (hVFF) and bone marrow mesenchymal stem cells (BM-MSC) to vibratory stimulus. In order to study these effects, a bioreactor capable of vibrating two cell seeded substrates was developed. The cell seeded substrates contact each other as a result of the sinusoidal frequency, producing a motion similar to the movement of true vocal folds. Utilizing this bioreactor, hVFF and BM-MSC were subjected to 200 Hz vibration and 20% strain for 8 hours. Immunohistochemistry (Ki-67 and TUNEL) was performed to examine cell proliferation and apoptosis respectively, while semi-quantitative RT-PCR was used to assess extracellular matrix related gene expression. HVFF significantly proliferated (p = 0.011) when subjected to 200 Hz vibration and 20% strain, while BM-MSC did not (p = 1.0). A statistically significant increase in apoptosis of BM-MSC (p = 0.0402) was observed under the experimental conditions; however high cell viability (96%) was maintained. HVFF did not have significantly altered apoptosis (p = 0.7849) when subjected to vibration and strain. Semi-quantitative RT-PCR results show no significant differences in expression levels of collagen I (BM-MSC p = 0.1951, hVFF p = v0.3629), fibronectin (BM-MSC p = 0.1951, hVFF p = 0.2513), and TGF-β1 (BM-MSC p = 0.2534, hVFF p = 0.6029) between vibratory and static conditions in either cell type. Finally, smooth muscle actin mRNA was not present in either vibrated or static samples, indicating that no myofibroblast differentiation occurred for either cell type. Together, these results demonstrate that BM-MSC may be a suitable alternative to hVFF for vocal fold tissue engineering. Further investigation into a larger number of gene markers, protein levels, increased number of donors and vibratory conditions are warranted.

## Introduction

The unique mechano-environment of vocal fold mucosa includes its ability to sustain oscillation up to 1000 hertz, amplitudes of 1 mm, and accelerations of 200–300 G [1], [2]. This tissue is highly adapted to meet the biomechanical requirements of everyday communication which can include phonatory vibration times of approximately 30% of every hour for heavy voice users; bioengineering strategies are needed for tissue that is not able to meet these needs. Because mechanical activity plays an important role in organogenesis during embryonic development it is predicted that a physiologic level of force is needed for the development of biomimetic engineering tissue constructs where mechanical functions are critical, such as the vocal fold mucosa [3]. Development of a novel bioreactor that provides a controllable, mechanically active environment will allow for the study of cellular responses to those mechanical conditions common to the native vocal mucosa native tissue environment and could also provide appropriate physical stimulation to bioengineered constructs for successful develop of functional tissue.

One of the biggest obstacles in tissue engineering of the vocal fold lamina propria is the lack of knowledge regarding the cellular response to relevant mechanical stimuli, primarily vibration. During phonation, the lamina propria regularly experiences vibrations greater than 120 Hz, a condition not seen anywhere else within the human body [4]. Vocal fold mucosa may experience extracellular and intercellular changes resulting in altered gene and/or protein expression as a result of the vibration exposure, as it is widely accepted that cells are inherently sensitive to their surroundings. It has previously been suggested that fibroblasts contain the necessary intracellular machinery to remodel the extracellular matrix (ECM) in response to mechanical stimulation [5]. In other parts of the body, fibroblast expression levels of extracellular glycoproteins have a direct correlation to applied mechanical stress [6]. Dermal fibroblasts have also been shown to increase collagen type I and fibronectin synthesis in response to mechanically induced strain [7]. A similar effect is observed in ligament fibroblasts, which secrete collagen I and III when subjected to axial strain [8]. Cardiac fibroblasts are also known to remodel their ECM in response to mechanical stimuli [9]. Laryngeal fibroblasts have been shown to increase mRNA levels of several ECM genes, both proteins and proteases, following mechanical vibration [10]. Recent work by Wolchok et al. support this finding by showing that laryngeal fibroblasts not only upregulate ECM related mRNA, but also secrete matrix proteins, including collagen I and fibronectin, when exposed to mechanical vibration [11]. It is unknown if human vocal fold fibroblasts (hVFF), the cells that are responsible for the production of the extracellular matrix of the vocal fold mucosa [12], respond to vibration in a similar fashion to remodel the ECM as fibroblasts from other locations in the larynx. An understanding of how hVFF alter ECM production and degradation in response to vibration is crucial for learning how to direct tissue growth in a future bioengineered vocal fold mucosa replacement.

In the development of a biomimetic tissue engineering construct for the vocal fold lamina propria, use of hVFF has apparent advantages given they are the native cell; however, healthy hVFF are unavailable commercially and primary cells are very difficult to acquire for transplantation. It is therefore necessary to investigate the suitability of other cell types for transplantation as measured by their response to mechanical forces that are common to the vocal fold lamina propria. Mesenchymal stromal cells originally isolated from bone marrow (BM-MSC), possess the ability to differentiate along multiple tissue lineages, participate in tissue repair and regeneration through a variety of paracrine mechanisms and suppress activation and proliferation of immune and inflammatory cells [13]. Hanson et al. have determined that hVFF indeed exhibit functionally similar characteristics to BM-MSC as defined by cell surface markers, differentiation potential, and immunophenotype [14]. The similar characteristics of these two cell types may designate BM-MSC as a viable cell for vocal fold tissue engineering. In addition to functional similarity, the benefits of using BM-MSC include their capacity for significant cell expansion ex vivo and their immunosuppressant properties. The availability of relatively large populations of these cells within adult bone marrow makes them accessible, efficiently obtained and easily expanded *ex vivo*. The availability of these cells within adults highlights their potential autologous use. There is also some evidence indicating that BM-MSC can suppress elements of the immune system, such as T-lymphocytes and B-lymphocytes [15]. These properties could identify BM-MSC as an optimal cell source for vocal fold tissue engineering.

Our objective of this work was to develop a custom bioreactor that vibrates a synthetic extracellular matrix (sECM) scaffold in which cells are seeded, allowing for the characterization of the functional phenotype of hVFF and BM-MSC. This characterization will be valuable in identifying an optimal cell source for future implantable tissue engineered scaffolds.

## Materials and Methods

### 1.1. Bioreactor Design

**Figure 1:**
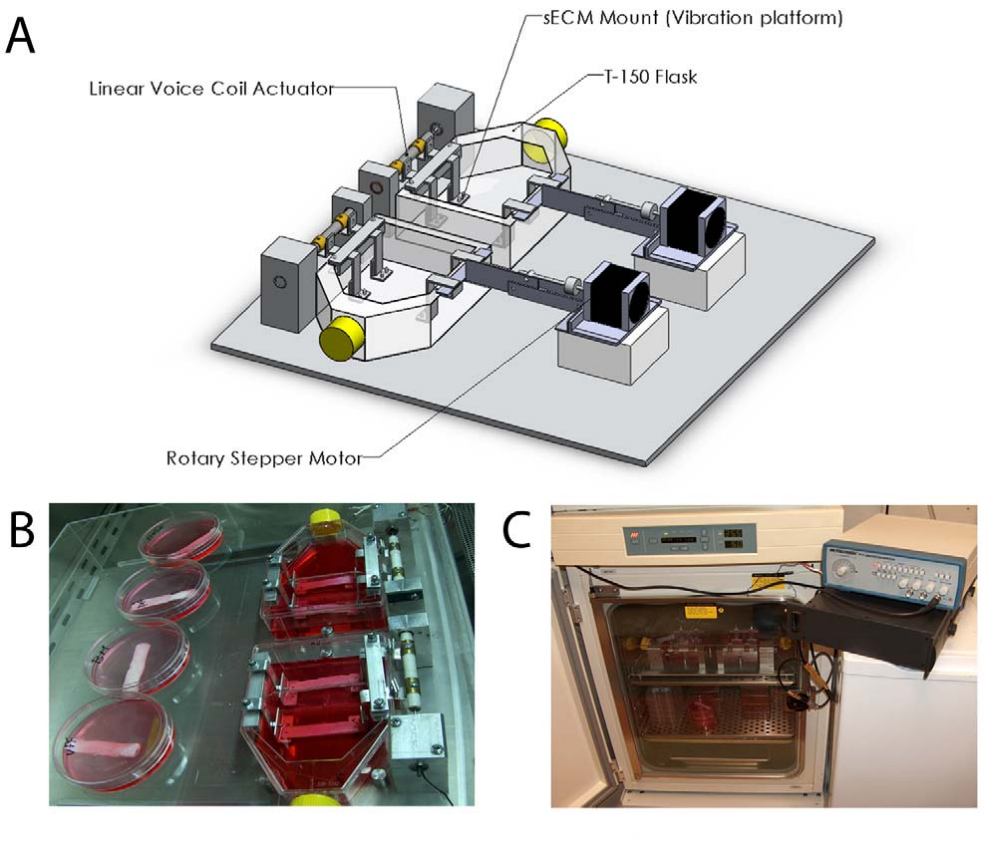
Schematic of the developed bioreactor. **A.** Bioreactor including T-flask, substrate, voice-coil actuator, linear stepper motors, rotary stepper motors, and scissor bars. **B.** Experimental setup, with static attachment in place of stepper motors. Non-vibrated controls can also be seen. **C.** Bioreactor within incubator. Wave-form generator is next to the incubator, sitting on top of the power amplifier.

Our current model of bioreactor simulates the vocal fold *in vivo* environment by subjecting cell-seeded synthetic extracellular matrix (sECM) strips to three stimuli – vibration, tensile stress, and dynamic angle change. sECM strips are mounted in a replaceable, sterile T-150 cell culture flask, which has an open upper surface, fastened to the bioreactor base (Figure 1). Each flask contains four cell-seeded sECM strips arranged in two pairs of one strip each, fastened to the bioreactor by protruding pins. Strips are attached at one end to the vibration platform, and at the other end to the aluminum scissor bars, allowing the strips to be subjected to all the stimuli types simultaneously. The bioreactor employs a different motor for each one of the three stimuli: a linear voice-coil actuator for vibration, a linear stepper motor for stretch, and a rotary stepper motor for angle change. The linear voice-coil actuator (H2W Technologies, Santa Clarita CA), encased by aluminum blocks, produces vibration through the use of a wave-form generator. The frequency signal, generated through custom software controlling an arbitrary wave form generator (USBDA128A, ACCES I/O Products, San Diego, CA), is amplified by a power amplifier (Samson Technologies, Hauppauge NY) before reaching the actuator. The linear actuator has an operating range of 0–2727 Hz using a sinusoidal waveform signal, and is controlled by input from a custom program controlling the wave-form generator. The actuator, when activated, undergoes purely horizontal translation. This results in the strip edges in each pair contacting each other in a manner similar to *in vivo* movement. Previous vocal fold bioreactors cause cell-seeded strips to vibrate, but not to contact each other [10], [11], [16].

Linear stepper motors (H2W Technologies, Santa Clarita, CA), attached to the long rails (platens) in back, stretch the sECM. When activated, the linear stepper motor undergoes pure translational motion up or down the rail, with a maximum displacement of six inches. This translation produced varying tension in the strips, depending on the length and thickness of the substrate. The linear stepper motor is controlled by programming the desired time duration and degree of stretch to the motor through computer-based software.

Rotary stepper motors (Automation Direct, Cumming, GA) mounted to the top of the linear motors altered the angle between each cell-seeded strip in a pair. The shaft of the motor rotated in such a way that the ends of the strip attached to the scissor bar moved further apart, while the ends attached to the vibration platform stayed at the same distance, effectively creating an angle between the strips. This angle can vary from 0–39 degrees, and is controlled by computer-based software.

For the purposes of this study, only the vibrational stimulus with strain was utilized. In order to accommodate this change, the scissor bar, linear stepper motors, and rotary stepper motors were removed. A new setup was made which uses the rotary stepper motors to control strain. Rotation of each motor causes the strips to be stretched in the axial direction (Figure 1B).

### 1.2. Fabrication of cellular scaffold

Tecoflex® SG-80A (6.75 g, Thermedics, Wickliffe, OH) was dissolved in dimethylacetamide (39.1 ml, Fisher Scientific, Pittsburgh, PA) overnight at 60°C. The temperature of the solution was increased to 80°C and Pluronic 10R5 (18.95 ml, BASF, Freeport, TX) was added slowly dropwise to the solution over the course of four hours. The temperature of the polymer solution was lowered to 60°C and pipetted into custom Delrin molds having dimensions of 65 mm long×15 mm wide×3.175 mm deep. The polymer-containing molds were placed at −20°C for 24 hours. The molds were placed in sterile, endotoxin-free water for 48 hours with four water changes. The design of the molds was such that the all sides of the polymer strip were soaked equally. Finally, the strips were dried by a 24-hour lyophilization. The final dimensions of the substrates were 50 mm×10 mm×2 mm.

### 1.3. Cell Culture

For all experiments, two different cell lines were used. Immortalized human vocal fold fibroblasts [17] were used from passage 9–15. The hVFF were thawed, plated separately in T-175 flasks, and grown in DMEM with 10% FBS, 1% Penicillin/Streptomycin, and 1% non-essential amino acids. BM-MSC, isolated from a single patient [15], were used from passage 5–10. The frozen cells were thawed, plated in a Corning HyperFlask (Corning, Corning, NY), and grown in α-MEM with 10% heat-inactivated FBS, 1% L-glutamine, 1% Penicillin/Streptomycin, and 1% non-essential amino acids. For all cell types, the confluent flasks were disassociated with .25% trypsin and 1 mM EDTA in HBSS, centrifuged and replated in separate Corning HyperFlasks. For immunohistochemistry analysis, cells were seeded into the three-dimensional substrates at a cell density of 8 million cells/substrate. For PCR analysis, cells were seeded into the three-dimensional substrates at a cell density of 4.75 million cells/substrate. To encourage cell attachment to the polyurethane scaffold, the scaffolds were soaked overnight in 20 µg/ml human fibronectin in phosphate buffered saline (PBS). The cell-seeded substrate strips were then immersed in their respective cell-culture media. All cell culture materials were obtained from Sigma-Aldrich, unless otherwise mentioned.

### 1.4. Cell seeding of 3-dimensional porous substrates

3D substrates were cold ethylene oxide sterilized and immersed overnight 20 µg/ml human fibronectin in PBS. Confluent HyperFlasks of human vocal fold fibroblasts were dissociated with .25% Trypsin and 1 mM EDTA in HBSS, centrifuged and resuspended in cell culture media. The cells were counted in a hemocytometer and resuspended for a final concentration of 1.8×10^6^ cells/mL in 10 mL of cell culture media. This cell-containing media was added dropwise to the surface of two strips and cultured in an incubator (37°C, 5% CO^2^) to allow cell attachment to the Tecoflex® scaffold. One substrate was transferred to the bioreactor where it was subjected to eight hours of vibration at 20% strain, while the other was placed in a sterile culture dish containing cell culture media, to be used as a non-vibrated control.

### 1.5. RNA extraction

Cells were dissociated from the sECM substrate by submerging the strip after vibration in 3 mL of .25% Trpysin, and kneading the strip with a plastic pestle to remove the cells. The cell solution was then centrifuged, and the supernatant removed. Total RNA was extracted as directed, using a Quiagen RNeasy mini kit (Qiagen, Valencia, CA), including the optional on-column DNase digestion.

### 1.6. cDNA synthesis

RNA was quantified using a Nanodrop-1000 Spectrophotometer (Thermo Scientific, Wilmington DE) and 200 ng was used for cDNA synthesis with Omniscript Reverse Transcriptase (Qiagen, Valencia, CA) and random hexamers (Integrated DNA Technologies, Coralville, IA). Reactions were performed in a Peltier Thermal Cycler PTC-200 (Roche, Indianapolis, IN) at 37°C for 1 hour. cDNA was quantified using the nanodrop and stored at 4°C for further PCR evaluation.

### 1.7. Gel Electrophoresis and Semi-Quantitative PCR

Amplification of cDNA was performed using the Peltier Thermal Cycler and cDNA specific primers (Integrated DNA Technologies, Coralville, IA). Primers were designed using NCBI BLAST for low melting temperature and specific cDNA amplification (Table 1). All primers were synthesized by Integrated DNA Technologies (Coralville, IA). The targets measured include collagen I–α1 (CIα1), fibronectin (FN), transforming growth factor beta (TGF-β1), and α-smooth muscle actin (SMA). Semi-quantitative PCR was carried out in single target reactions, with the reference gene Human Ribosomal Protein 14 (hRPS14) used for all targets. Non-template reactions were used as control for negative amplification of target. PCR products were separated on a 2% SeaKem LE Agarose (Cambrex Bioscience, Rockland, ME) gel stained with .1% ethidium bromide (Bio-Rad, Hercules, CA). Each agarose gel was subjected to 50 V of DC current for 1 hour. Fluorescent images of gels were obtained with an Ultraviolet Transilluminator (UVP LLC, Upland, CA). Densitometry was performed on gel images using Metamorph imaging software (Molecular Devices, Downington, PA).

**Table 1:** Primer sequences and product sizes for RT-PCR.

Target	GeneID	cDNA Forward Primers	cDNA Forward Primers	Size of PCR Product (bp)
**Human**	6208	5′-	5′-CTGCGAGTGCTGTCAGAGG-3′	157
**Ribosomal**		TCACCGCCCTACACATCAAACT-		
**Protein 14**		3′		
**Fibronectin**	2335	5′-GTGGGAGTTGGGCTGACTCG	5′-TGAAGAGGGGCACATGCTGA-3′	274
		-3′		
**Transforming**	7040	5′-	5′-	126
**Growth Factor-β**		TGCTCGCCCTGTACAACAGCA-3′	CGTTGTGGGTTTCCACCATTAGCA	
			-3′	
**Collagen Iα1**	1277	5′-	5′-CCTTCTTGAGGTTGCCAGTC-3′	360
		CGATGGATTCCAGTTCGAGTA-3′		
α**-Smooth** **Muscle Actin**	59	5′-TGAGACTTTCAATGTCCCAGC-3′	5′-ACGCTCAGTCAGGATCTTCA-3′	200

### 1.8. Immunofluorescent histological analysis

One half of each cell-seeded substrate was fixed for histological analysis with glyoxal Prefer (Anatech LTD, Battle Creek, MI) overnight at room temperature. Samples were immersed in 30% sucrose overnight, after which they were embedded in optimal cutting temperature (OCT) embedding compound (Sakura Finetek, Torrance, CA) and frozen in a dry ice/ethanol bath. Each substrate was serially sectioned at 10 µm with a Cryostat and transferred to microscope slides (Superfrost Excell, Fisher Scientific). The slides were stained with antisera directed against Ki-67 (1∶50, Fisher Scientific) and visualized with Tetramethyl Rhodamine Isothiocyanate (TRITC). Separate slides were stained with an *in situ* cell death detection kit (Roche Applied Science, Indianapolis, IN) to identify apoptosis.

Cell nuclei for all slides were identified with DAPI (1∶10000, Sigma). Slides were analyzed at 20× magnification using a fluorescence microscope (E600, Nikon). All images of fluorescence were taken with a digital camera (DP71, Olympus). All fluorescent images were converted to 16-bit TIFF format for analysis in ImageJ (NIH). All cell nuclei, as well as Ki-67 and apoptosis markers, were quantified using Metamorph imaging software (Molecular Devices, Downington, PA). In order to count the number of cells in each image, the count nuclei function was used. The count nuclei function was also used to count the number of apoptotic cells, by employing length and area parameters obtained from positive controls. To quantify which cells were proliferating, a binary mask was created with the fluorescent image for Ki-67. The mask was overlaid with the DAPI image, and cell nuclei containing Ki-67 were counted manually. Ten percent of the images were measured again for intrarater reliability (*P* = 0.21, paired *t* test)

### 1.9. Statistical Analysis

Immunofluorescent histological markers, specifically TUNEL staining and Ki-67, normalized to the number of observed cells per image, were compared statistically by Chi-square test and Fishers exact test, where p<0.05 was considered significant. Comparison was made between the overall numbers of vibrated cells to non-vibrated cells. PCR gel images were compared statistically between vibrated and non-vibrated conditions using a Student t-Test. The intensity of all bands was normalized to the ribosomal protein house keeping gene hRPS14. Findings were considered significant at p<.05.

## Results

### 2.1. Immunohistochemistry

**Figure 2:**
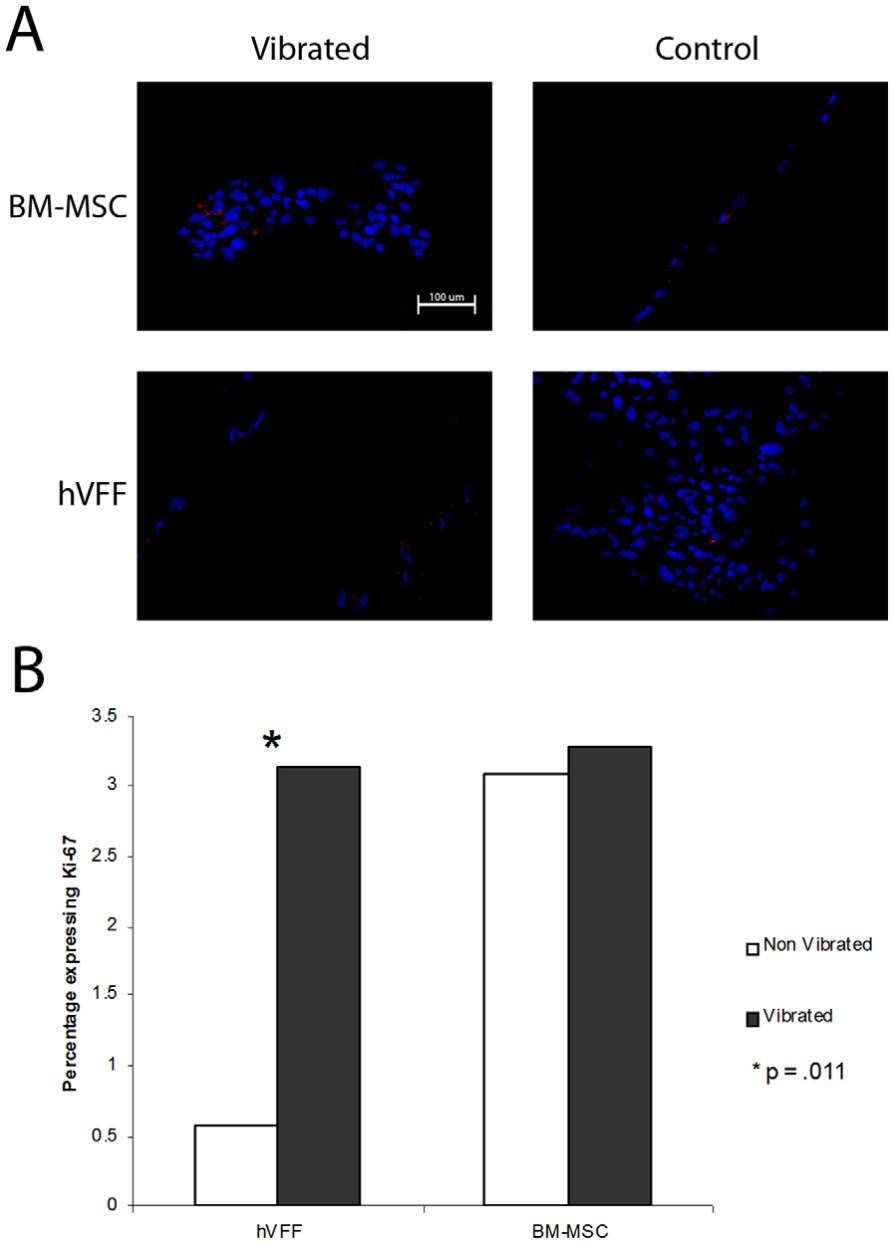
Proliferation of hVFF and BM-MSC. **A.** Representative 20× of vibrated and control immunohistochemistry for each cell type. Proliferating cells are marked by TRITC (red), cell nucleus (blue DAPI). **B.** Percentage of proliferating cells under each condition. An increase in proliferation is observed with vibrated hVFF compared to controls. *(p = .011).

**Figure 3:**
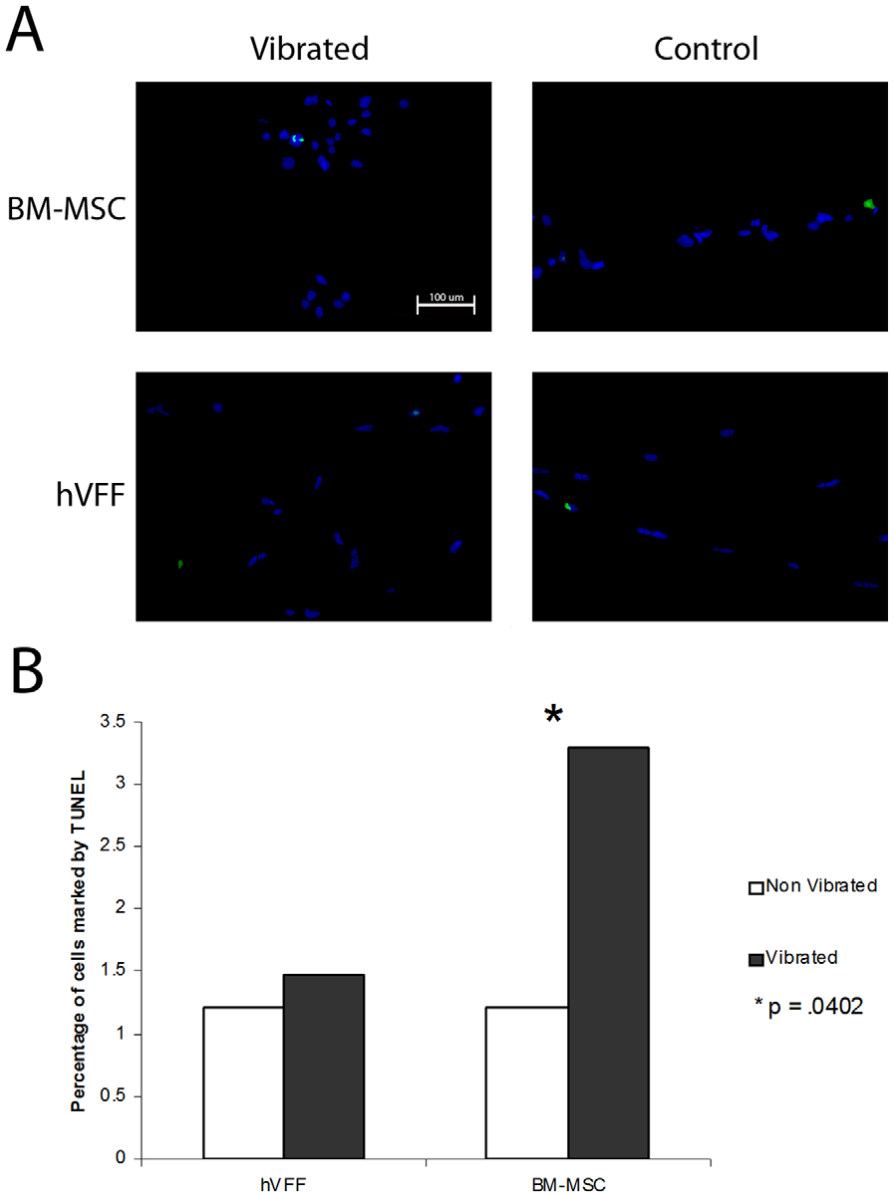
Apoptosis in hVFF and BM-MSC. **A.** Representative 20× pictures of vibrated and control immunohistochemistry sections for each cell type. Apoptotic cells are marked by FITC (green), cell nuclei DAPI (blue). **B.** The percentage of proliferating cells under each condition. An increase in apoptosis is observed with vibrated BM-MSC compared to controls. * (p = .0402).

Fluorescent staining demonstrated that Ki-67, a cellular marker of proliferation, was measured in less than 5% of cells for each cell type and condition (Figure 2A). Proliferation significantly increased in vibrated hVFF compared to non-vibrated controls (p = .0111, Figure 2B). No significant differences were measured for BM-MSC vibrated compared to non vibrated (p = 1.0). Apoptosis, as measured by a TUNEL assay, was detected in less than 6% of cells in each cell type and condition (Figure 3A). A statistically significant increase in apoptosis over controls was measured for BM-MSC (p = .0402, Figure 3B) but not for hVFF (p = .7849). Moreover, cells that detached from the sECM into the media during the experiment showed characteristics of apoptosis such as condensation and shrinkage of the nucleus by light microscopy [18] (data not shown). Large regions of cell death were not detected anywhere on the substrate sections for either of the cell types.

### 2.2. Gene expression analysis

**Figure 4:**
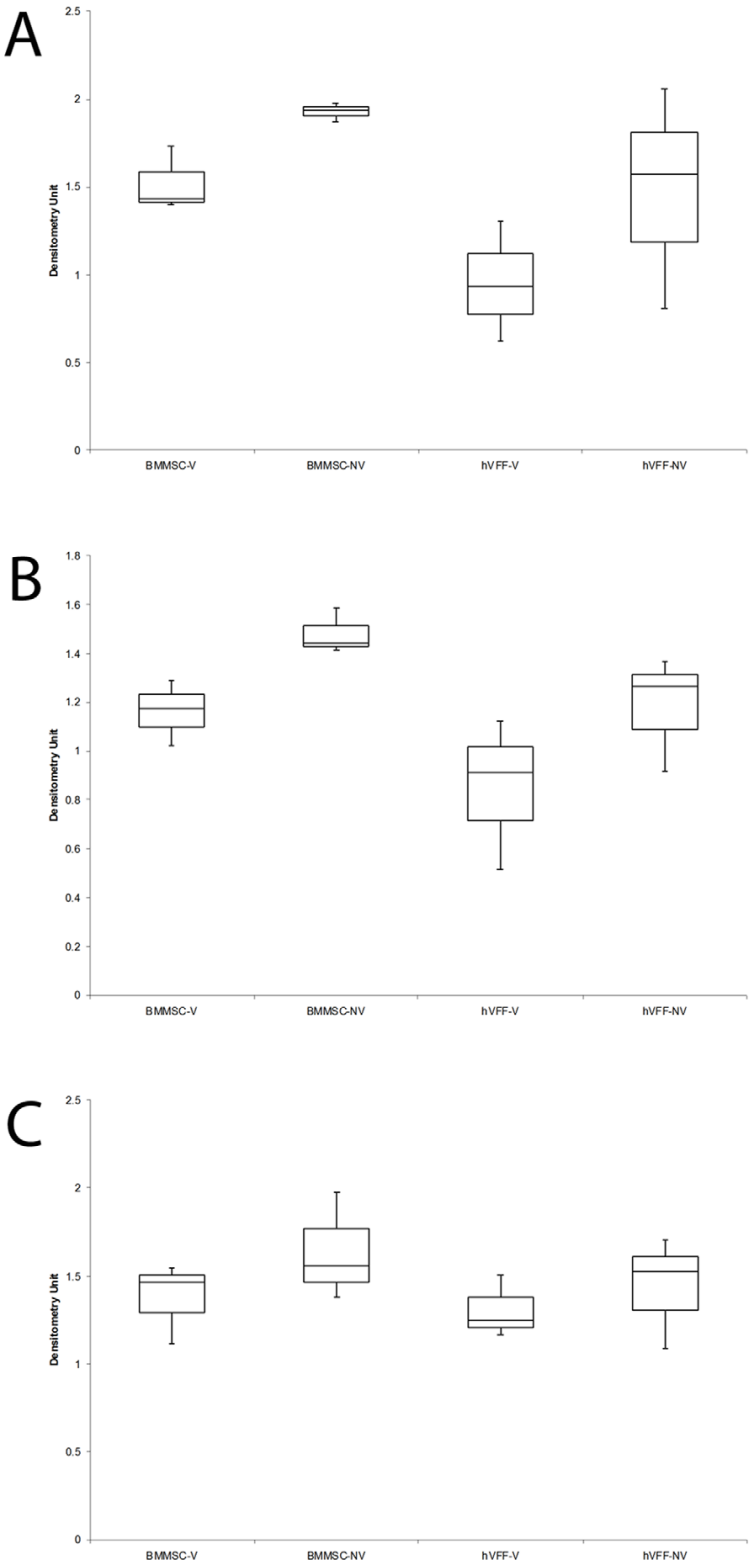
Gene Expression for hVFF and BM-MSC. **A.** Box and whisker plot showing expression changes for CIα1. No statistical difference was found between vibrated and control for either cell type. **B.** Box and whisker plot showing expression changes for FN. No statistical difference was measured between vibrated and control for either cell type. **C.** Box and whisker plot showing expression changes for TGF-β1. No statistical difference was measured between vibrated and control for either cell type.

Analysis performed following PCR and gel electrophoresis targeting CIα1 demonstrated no significant difference between vibrated and non-vibrated cells in either BM-MSC (p = .1951) or hVFF (p = .3629) (Figure 4A). No statistically significant differences were measured for FN between vibrated and non-vibrated controls in either cell type (BM-MSC p = .1951, hVFF p = .2513)(Figure 4B). SMA expression, a marker characteristic of myofibroblasts, was not measured in either condition for BM-MSC or hVFF (data not shown). Expression of the secreted factor TGF-β1 is not significantly different for any condition (Figure 4C). No significant differences were found between vibrated and non-vibrated cells in either BM-MSC (p = .2534) or hVFF (p = .6029).

## Discussion

Successful development of new therapeutic regenerative interventions for vocal fold disease, in addition to an improved understanding of molecular development, pathogenesis and biological features of the vocal fold lamina propria extracellular matrix (ECM) depend on the availability of reproducible *in vitro* models. Bioreactors are laboratory tissue culture devices that provide a controllable, mechanically active environment that can be used to study and potentially improve engineered tissue structure, properties and integration. The use of bioreactors has been successful in regeneration of bone, vascular and cardiac tissue [19]–[21]. The development and optimization of a unique bioreactor can be utilized to address the fundamental question of how mechanical modulation affects cell behavior and its production of ECM specific to the vocal fold. This knowledge will allow for the development of an ideal engineered cell seeded vocal fold construct.

Cell sourcing in the development of a biomimetic construct for the vocal fold is problematic. A distinct cell source of healthy native human hVFF does not exist. Normal vocal fold tissue from live donors is virtually impossible to obtain, and when they are obtained, insufficient numbers of cells are more often then not cultivated. Six immortalized hVFF lines have been developed, banked and have been used for in vitro investigation [23]; immortalized lines are not an ideal universal donor line. Rather to be successful there needs to be the availability of distinct cell sources for tissue regeneration, cell characterization and development of universal donor cell lines [22]. BM-MSC have been utilized in other areas of tissue engineering [23]–[25] and given their regenerative potential and similarity to hVFF, comparisons between their behaviors in a developed bioreactor were made.

BM-MSC and hVFF had similar gene expression results such that vibration did not induce up or down regulation of collagen 1 or Fn. This is important as it shows that extended vibration may not cause hVFF to build up the ECM, creating a more fibrotic vocal fold mucosa. The lack of upregulation of these ECM proteins in BM-MSC is also important, as it indicates that they could potentially be placed within vocal fold tissue without contributing to fibrosis. Our results do differ from previously published vibration reports that have subjected laryngeal fibroblasts to strain and vibration [10], [11]. This discrepancy could occur as the result of several phenomena. hVFF were assessed in the present study, whereas laryngeal fibroblasts, more specifically tracheal scar fibroblasts have been used in other reports. It is likely that fibroblasts from different parts of the body respond differently to the same stimuli as fibroblasts from distinct anatomical sites show variable levels of expression from large groups of expressed genes [26]. Vibration frequency and duration is also markedly different between studies. Lastly, other versions of vibrational bioreactors only vibrate one strip whereas the current bioreactor vibrates two strips that contact each other as a result of the sinusoidal frequency. It is likely that one or a combination of these differences caused the observed difference in results.

Evidence of SMA expression was not observed in any cell type indicating that neither BM-MSC nor hVFF display a myofibroblast phenotype following vibration [27]. Myofibroblasts are present in nearly all fibrotic states, and are an important part of the wound healing response. The fact that these cells do not exhibit myofibroblast characteristics would seem to indicate that 8 hours of constant vibration does not elicit a strong wound healing response. Previous vocal fold bioreactor studies have not examined TGF-β1 expression levels due to strain or vibration. Our results show that though hVFF and BM-MSC both constitutively express mRNA for TGF-β1, there is no evidence that vibration increases expression in either cell type. This finding is also consistent with CIα1 findings, as increased levels of TGF-β1 has been shown to increase collagen secretion [28]. TGF-β1 treatment is also associated with differentiation towards a myofibroblast phenotype in hVFF. The lack of myofibroblast markers further supports this finding.

Differences were found in regards to proliferation and apoptosis between the two cell types. As indicated by the TUNEL assay, high cell viability (96%) was maintained for both hVFF and BM-MSC, yet BM-MSC had significantly more apoptotic cells. It should be noted that less than 4% of BM-MSC in the vibrated condition underwent apoptosis after 8 hours of constant vibration. This difference may not be of strong impact as 8 hours of constant vibration is far longer than is typical for heavy voice users [29]. Our immunohistochemistry data indicate that the sustained vibration condition leads to an increase of fibroblast proliferation. Previous studies have found that externally applied static strain can induce proliferation in a variety of cells, including fibroblasts [30]. In these pathways, the MAPK/ERK pathway is activated due to receptor stimulation at focal adhesions. In particular, ERK2 has shown to be activated simply due to stretch on fibronectin [31]. Given this evidence, there is potential that this pathway was activated in the vibrated hVFF. Further study is needed to further discern the effects of applied vibration and strain.

Several limitations of our investigation warrant discussion. For the hVFF, an immortalized cell line was used instead of primary cells. It is possible that the immortalized cells would respond differently than primary cells, although previous work indicates that the response should be similar [17]. Only one cell line for each cell type was used, and as such each cell type only came from one respective donor. The limited number of donors does not allow the data to show if the results differ between individuals. The differences in BM-MSC response between donors for other fields is documented [32], and it stands to reason that some difference may be seen here as well. In addition, only one vibration pattern was used (i.e, continuous vibration for 8 hours) in the present study. In order to more accurately quantify the effects of vibration, more than one paradigm needs to be used. A pattern more consistent with typical speech, such as vibrate for 30 seconds, rest for 10 seconds, repeated for 8 hours may provide insight into the effects of a normal day of speaking on these cell types. The frequency, 200 Hz, is also closer to the female voice range as compared to the male range. It is possible that a different response may have been seen at a lower frequency, such as 110 Hz, closer to a typical male speaking voice. Finally, a limited number of genes were investigate in this study. Though no difference was seen following vibration with the chosen ECM genes, the expression of other genes may have changed. The number of gene targets should be expanded to further determine the changes, or lack thereof, following vibration.

### Conclusions

A newly developed vibration bioreactor, capable of causing two cell seeding substrates to contact each other in a wave-dependent motion, was designed and tested. Mechanical forces applied to BM-MSC and hVFF did not yield significant gene expression differences after 8 hours of vibration. BM-MSC may be an ideal alternate for the development of a bimimetic tissue engineered construct for vocal folds. Future studies will investigate the effects of vibration using more donors, while also investigating more protein and gene markers.
